# Author Correction: Factors of the bone marrow microniche that support human plasma cell survival and immunoglobulin secretion

**DOI:** 10.1038/s41467-019-08400-0

**Published:** 2019-01-17

**Authors:** Doan C. Nguyen, Swetha Garimalla, Haopeng Xiao, Shuya Kyu, Igor Albizua, Jacques Galipeau, Kuang-Yueh Chiang, Edmund K. Waller, Ronghu Wu, Greg Gibson, James Roberson, Frances E. Lund, Troy D. Randall, Iñaki Sanz, F. Eun-Hyung Lee

**Affiliations:** 1 Division of Pulmonary, Allergy, Critical Care & Sleep Medicine, Emory University, Atlanta, GA USA; 2 School of Biological Sciences, Georgia Institute of Technology, Atlanta, GA USA; 3 School of Chemistry and Biochemistry, Georgia Institute of Technology, Atlanta, GA USA; 4 Department of Human Genetics, Emory University, Atlanta, GA USA; 5 Department of Medicine & University of Wisconsin Carbone Cancer Center, University of Wisconsin in Madison, Madison, WI USA; 6 Division of Hematology & Oncology, University of Toronto, Toronto, ON Canada; 7 Pediatrics & Hematology/Oncology, Emory University, Atlanta, GA USA; 8 Department of Orthopedics, Emory University, Atlanta, GA USA; 9 Department of Microbiology, University of Alabama at Birmingham, Birmingham, AL USA; 10 Division of Clinical Immunology & Rheumatology, University of Alabama at Birmingham, Birmingham, AL USA; 11 Division of Rheumatology, Emory University, Atlanta, GA USA; 12 Lowance Center for Human Immunology, Emory University, Atlanta, GA USA

Correction to: *Nature Communications*; 10.1038/s41467-018-05853-7; published online 12 September 2018

The original version of this Article omitted a declaration from the Competing interests statement, which should have included the following: ‘A patent has been applied for by Emory University with F.E.L., I.S., and D.C.N. as named inventors. The patent application number is PCT/US2016/036650’. This has now been corrected in both the PDF and HTML versions of the Article.

