# Supplementary material for: Factors of the bone marrow microniche that support human plasma cell survival and immunoglobulin secretion
Source: Nat Commun. 2018 Sep 12;9:3698. doi: 10.1038/s41467-018-05853-7 (PMC6135805; doi:10.1038/s41467-018-05853-7)
Supplement: Supplementary file 1 — Supplementary Information [file 41467_2018_5853_MOESM1_ESM.docx]

**Supplementary Information**

Factors of the Bone Marrow Microniche that Support Human Plasma Cell Survival and Immunoglobulin Secretion

Nguyen DC. et al.

**Supplementary Figure 1.** Single cytokines alone in R10 provide no pro-survival advantage.

**Supplementary Figure 2.** Addition of single cytokines in MSC secretome provides no pro-survival advantage.

**Supplementary Data 1.** The 556 overlapping targets, the 2,558 DEG, and the 4,426 potential PPI.

**Supplementary Data 2.** The 20 GSEA pathways.

**Supplementary Data 3.** The potential protein-protein interactions for fibronectin (FN-1) and YWHAZ.

**Supplementary Data 4.** The 10 GSEA pathways.


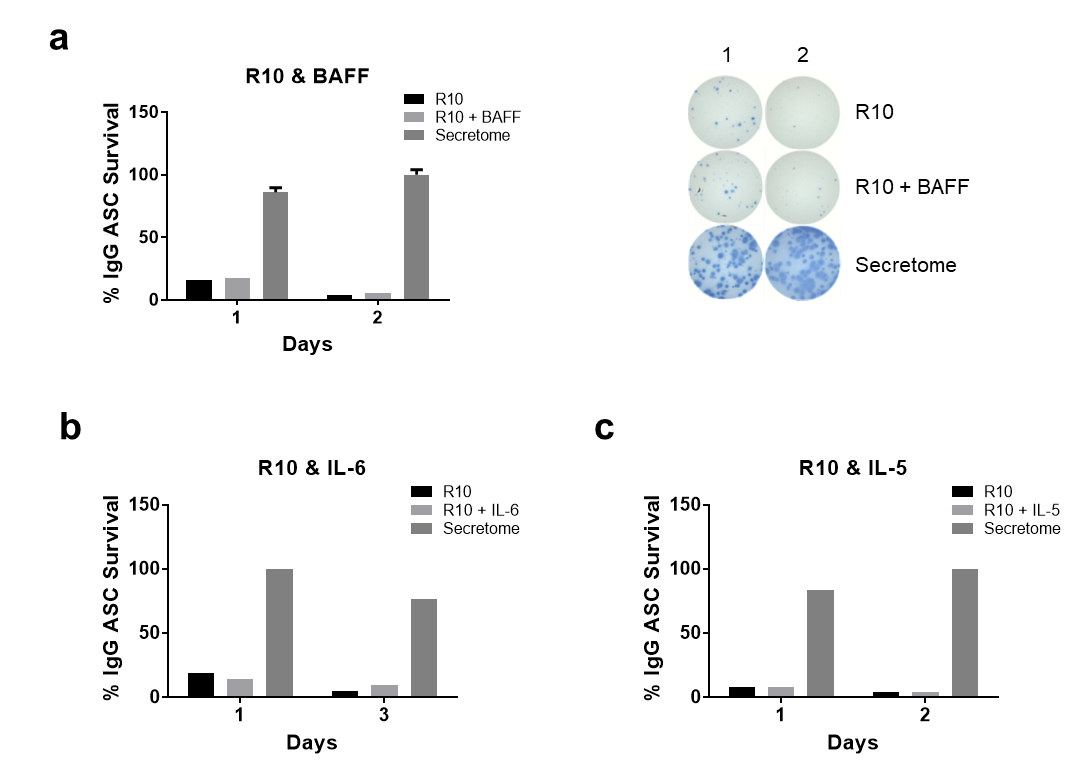


**Supplementary Figure 1****.** **Single cytokines alone in R10 provide no pro-survival advantage.** Addition of BAFF, IL-5, or IL-6 alone into R10 has no advantage on ASC *in vitro* survival. Blood ASC after vaccination were cultured in R10 alone, R10 plus (**a**) BAFF, (**b**) IL-6, or (**c**) IL-5, and MSC secretome alone (positive control). Percentage of IgG Elipsots normalized to maximal frequency on days 1-3. In (a), representative images of Elispot wells are shown on the right (numbers, day in culture). Representative of at least 2 experiments.


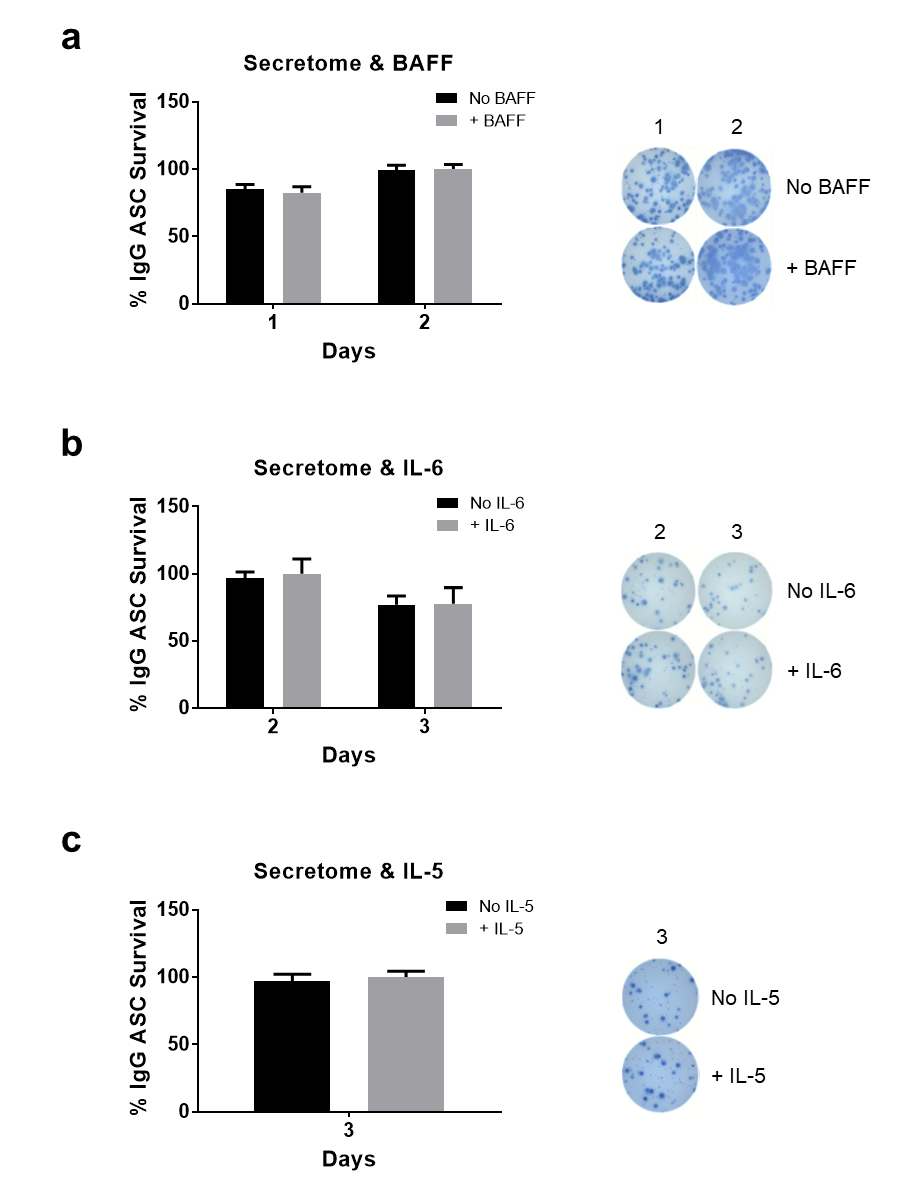


**Supplementary Figure 2.** **Addition of single cytokines in MSC secretome provides no pro-survival advantage** *(please see the legends on the following page).*


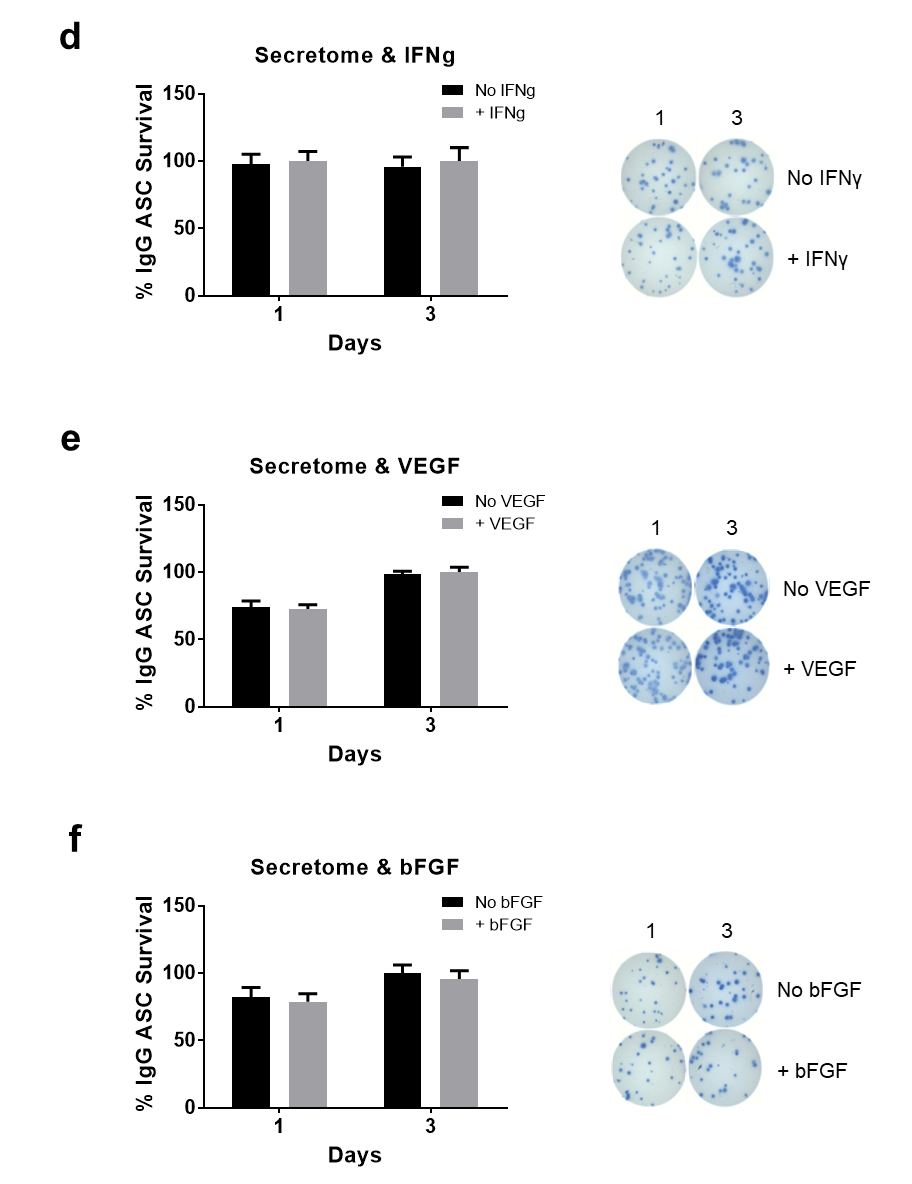


**Supplementary Figure 2.** **Addition of single cytokines in MSC secretome provides no pro-survival advantage.** Addition of single cytokines into the secretome has no advantage on ASC *in vitro* survival. Blood ASC were cultured in the secretome alone or with the addition of (**a**) BAFF, (**b**) IL-6, (**c**) IL-5, (**d**) IFNg, (**e**) VEGF, or (**f**) bFGF. Percentage of IgG Elipsots normalized to maximal frequency on days 1-3. Representative images of Elispot wells are shown on the right (numbers, day in culture). Representative of at least 2 experiments.
