# Supplementary material for: Factors of the bone marrow microniche that support human plasma cell survival and immunoglobulin secretion
Source: Nat Commun. 2018 Sep 12;9:3698. doi: 10.1038/s41467-018-05853-7 (PMC6135805; doi:10.1038/s41467-018-05853-7)
Supplement: Supplementary file 3 — Description of Additional Supplementary Files [file 41467_2018_5853_MOESM3_ESM.pdf]

Description of Additional Supplementary Files

File Name: Supplementary Data 1
Description: The 556 overlapping targets, the 2,558 DEG, and the 4,426 potential PPI.

File Name: Supplementary Data 2
Description: The 20 GSEA pathways.

File Name: Supplementary Data 3
Description: The potential protein-protein interactions for fibronectin (FN-1) and YWHAZ.

File Name: Supplementary Data 4
Description: The 10 GSEA pathways.
